# Pulmonary Vein Tumor Thrombus With Intracardiac Extension Secondary to Poorly Differentiated Bronchogenic Carcinoma

**DOI:** 10.7759/cureus.8278

**Published:** 2020-05-25

**Authors:** Saif Faiek, Ishita Malik, Rhea Farquhar, Vikram Lal, Aditya Bansal

**Affiliations:** 1 Internal Medicine, AtlantiCare Regional Medical Center, Atlantic City, USA; 2 Internal Medicine/Pulmonary & Critical Care Medicine, AtlantiCare Regional Medical Center, Atlantic City, USA

**Keywords:** pulmonary vein thrombosis, pulmonary vein tumor thrombus, left atrial tumor thrombus

## Abstract

Pulmonary vein thrombosis (PVT) is a rarely encountered disease entity with varied clinical presentations. It has been reported to be associated with underlying lung malignancy in multiple case reports. Diagnosis can be challenging due to nonspecific symptoms on presentation. Herein, we report a 67-year-old male patient with a history of extensive smoking and chronic obstructive pulmonary disease (COPD) who presented with multiple hemoptysis episodes. CT scan of the chest with contrast showed multiple right lower lobe (RLL) lung masses and a thrombus in the inferior pulmonary vein. After various imaging modalities and transthoracic biopsy of the lung mass, the patient was diagnosed with pulmonary vein tumor thrombus secondary to poorly differentiated bronchogenic carcinoma with intracardiac extension. The patient was started on Eliquis for anticoagulation and is currently in the process of beginning chemo/radiation therapy for the underlying malignancy.

## Introduction

Pulmonary vein thrombosis (PVT) is a rare and underdiagnosed clinical entity. The majority of cases are associated with primary or metastatic lung malignancy and lung surgery -- lung transplantation or lobectomy [1]. Other associated conditions include: atrial fibrillation, radiofrequency ablation, polycythemia vera, blunt chest trauma, and idiopathic causes [2]. In those associated with lung malignancy, the exact incidence is still unknown due to limited data in literature, with only a handful of cases reported [3]. 

## Case presentation

**Figure 1:**
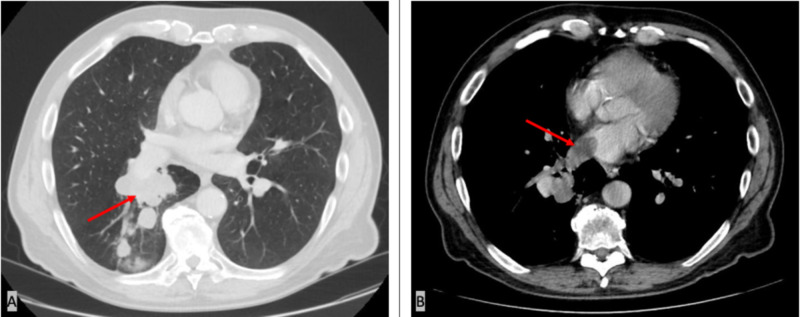
CT scan of the chest with contrast. A: showing multiple masses in the lower lobe of the right lung likely represent lung malignancy (red arrow); B: showing a clot likely representing a tumor thrombus in the left atrium extending from the right lower lobe (RLL) pulmonary veins (red arrow).

A 67-year-old male with a history of extensive smoking and chronic obstructive pulmonary disease (COPD) presented to the hospital for evaluation of multiple episodes of hemoptysis and abnormal findings on CT scan of the chest. His symptoms started a few months prior to presentation with a productive cough, for which he received several rounds of antibiotics prescribed by his primary care physician. His symptoms did not improve and progressed to hemoptysis. A chest X-ray showed a right suprahilar nodular density. CT scan of the chest with contrast was then ordered and revealed multiple masses in the right lower lobe (RLL) suspicious for lung carcinoma. A thrombus extending through the RLL pulmonary veins into the left atrium was also noted (Figure 1).

**Figure 2:**
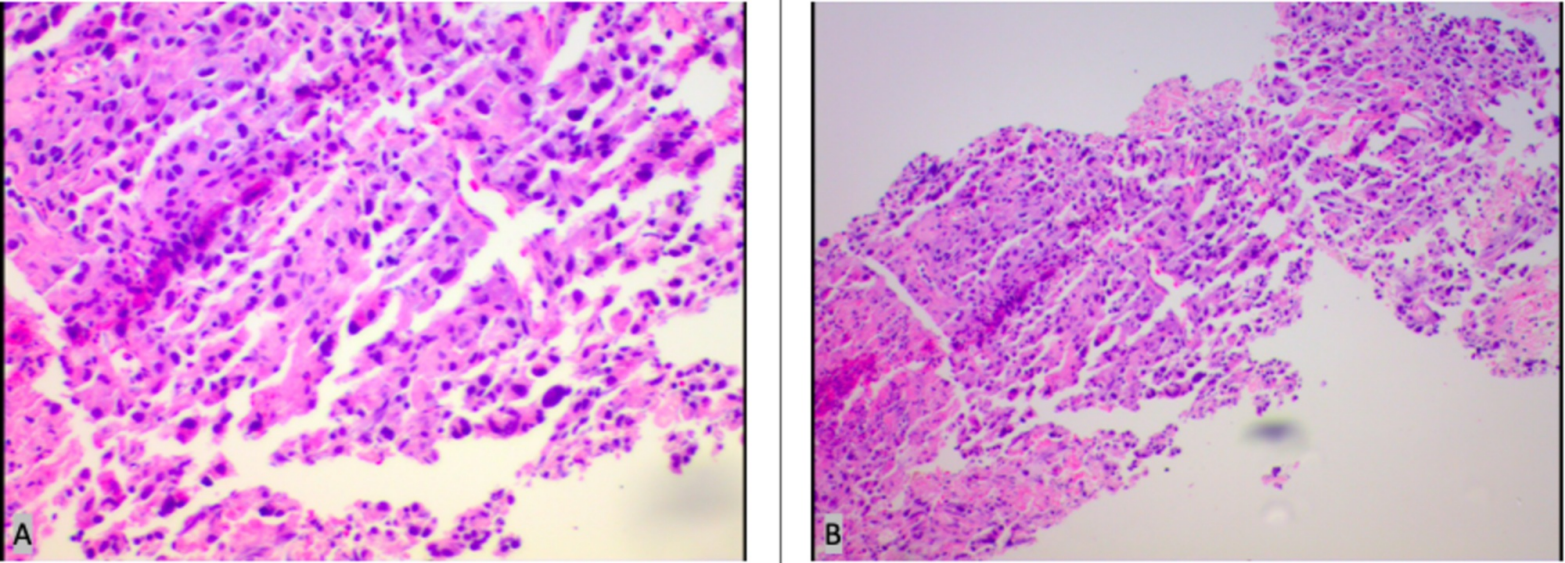
Trans-thoracic needle biopsy of the RLL mass. A: (H&E), 100x magnification; B: (H&E), 200x magnification. Both images are showing poorly differentiated tumor cells, which were only positive for CK7 and Caldesmon. All other immunohistochemical markers were negative. RLL, right lower lobe

Upon admission to the hospital, the patient was evaluated by pulmonary and oncology teams, and he was started on a heparin drip for anticoagulation. The patient underwent a trans-thoracic needle biopsy of the RLL mass, and pathology was consistent with the diagnosis of a poorly differentiated malignant neoplasm (Figure 2). Trans-thoracic echo performed during the patient’s hospital stay showed a poorly visualized left atrial mass. The patient was then discharged on Eliquis and instructed to follow-up with cardiology, pulmonology, and oncology specialists as an outpatient.

**Figure 3:**
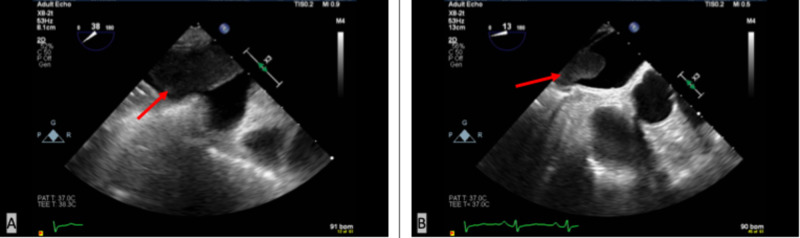
Trans-esophageal echocardiogram. A: a tumor thrombus extending through the right inferior pulmonary vein (red arrow); B: left atrial tumor thrombus (red arrow).

**Figure 4:**
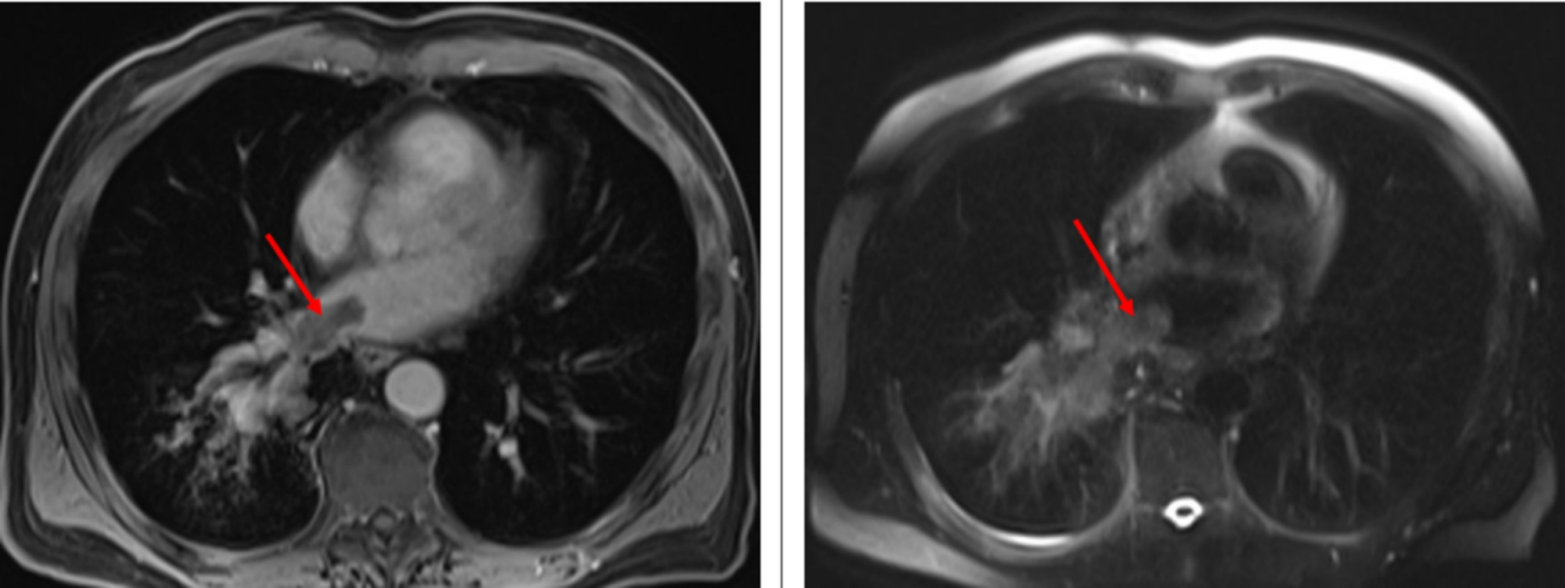
Cardiac MRI, with and without intravenous gadolinium demonstrates a tumor thrombus involving the right inferior pulmonary vein and extending into the left atrium (red arrow).

Trans-esophageal echo and cardiac MRI were done as an outpatient confirming a 1.7 cm x 1.4 cm x 1.3 cm left atrial tumor thrombus mass extending from the RLL lung mass through the right inferior pulmonary vein (Figures 3-4). Positron emission tomography (PET) scan and MRI of the brain ordered for complete staging, showed RLL masses and hypermetabolic precarinal and right paratracheal lymph nodes suspicious for nodal metastasis with no evidence of distant metastasis. The patient's lung cancer was classified as T4, stage IIIb, for which he will be started on chemo (carboplatin - taxol) and radiation therapy.

## Discussion

Pulmonary vein thrombosis is a rare yet potentially serious and life-threatening clinical condition. Most of the patients are asymptomatic or have nonspecific symptoms such as cough, hemoptysis, weight loss, and dyspnea as a result of pulmonary edema or infarction [3-4]. In cases associated with lung cancer the hypothesized underlying mechanism seems to involve either of the following: direct extension of the primary lung tumor into the pulmonary vein or mechanical compression over the pulmonary vein, as well as the prothrombotic state associated with the malignancy itself contributing to the formation of a thrombus [1, 4-5]. Complications, described previously in the literature that are associated with PVT include pulmonary infarction, pulmonary edema, systemic embolization (e.g. ischemic stroke, renal and splenic infarction) and sudden cardiac death due to inflow obstruction at the mitral valve [3, 6-7].

The diagnosis of PVT is usually challenging in the setting of nonspecific symptoms, and it is made based on the findings of multiple imaging modalities. Newer CT techniques have made identifying PVT possible in a similar manner to which pulmonary arterial emboli are detected. Using the pulmonary venous phase of contrast CT of the chest will show a filling defect in the pulmonary vein and sometimes an extension of the defect into the left atrium, similar to our case [1, 3]. Echocardiography also plays a role in the evaluation of PVT and the trans-esophageal modality is usually preferred due to precise visualization of the pulmonary vein and demonstration of possible PVT extension into the atrium [3, 8-9]. MRI imaging is another useful modality for diagnosis as it differentiates between tumor and bland thrombus in the pulmonary vein [1, 10].

There is no clear consensus regarding the treatment of PVT. The choice of therapy mainly depends on the clinical condition of the patient and the underlying etiology. In the absence of bleeding, anticoagulation is the mainstay of therapy to prevent clot progression and thrombus shedding. A literature review did not indicate the preferred duration of anticoagulation or a preference between oral vitamin K antagonist, low molecular or unfractionated heparin [3, 10].

In our case, the patient’s bronchogenic carcinoma extending through the right inferior pulmonary vein into the left atrium was classified as T4 and belongs to stage IIIb. In this type of malignancy, the addition of surgical resection to chemotherapy and radiotherapy treatment is still controversial, with a poor prognosis postoperatively. If radical resection is to be considered, it should be done under cardiopulmonary bypass to decrease the risk of systemic seeding or embolization of the tumor [11-12].

## Conclusions

With the advancement of various imaging modalities and their broad utilization, clinicians will likely face an increased rate of PVT detection and diagnosis. We describe a rare case of pulmonary vein tumor thrombus with left atrial extension secondary to bronchogenic carcinoma. PVT usually presents with nonspecific symptoms, which makes early recognition paramount for successful treatment of the patient and for preventing severe complications. Further studies and experience are required to establish standardized treatment protocols.
